# The profiling of microbiota in vaginal swab samples using 16S rRNA gene sequencing and IS-pro analysis

**DOI:** 10.1186/s12866-021-02149-7

**Published:** 2021-03-31

**Authors:** M. Singer, R. Koedooder, M. P. Bos, L. Poort, S. Schoenmakers, P. H. M. Savelkoul, J. S. E. Laven, J. D. de Jonge, S. A. Morré, A. E. Budding

**Affiliations:** 1 Laboratory of Immunogenetics, Department of Medical Microbiology and Infection Control, Amsterdam UMC, location VUmc, Amsterdam, the Netherlands; 2 Tubascan, Spin-off at the Department of Medical Microbiology and Infection Control, Amsterdam UMC, location VUmc, Amsterdam, the Netherlands; 3 Division of Reproductive Endocrinology and Infertility, Department of Obstetrics and Gynecology, Erasmus University Medical Center, Wytemaweg 12, Rotterdam, 3015 CN The Netherlands; 4 InBiome B.V, Amsterdam, The Netherlands; 5 Division Obstetrics, Department of Obstetrics and Gynecology, Erasmus University Medical Centre, Rotterdam, The Netherlands; 6 Department of Medical Microbiology, Maastricht University Medical Center, Maastricht, the Netherlands; 7 Department of Medical Microbiology and Infection Control, Amsterdam UMC, location VUmc, Amsterdam, the Netherlands; 8 ARTPred B.V, Seringenstraat 15, ‘s Hertogenbosch, 5213 GS The Netherlands; 9 Institute of Public Health Genomics, Department of Genetics and Cell Biology, Research Institute GROW, Faculty of Health, Medicine & Life Sciences, University of Maastricht, Maastricht, The Netherlands

**Keywords:** Vaginal microbiota, IS-pro, 16S rRNA gene sequencing

## Abstract

**Background:**

16S rRNA gene sequencing is currently the most common way of determining the composition of microbiota. This technique has enabled many new discoveries to be made regarding the relevance of microbiota to the health and disease of the host. However, compared to other diagnostic techniques, 16S rRNA gene sequencing is fairly costly and labor intensive, leaving room for other techniques to improve on these aspects.

**Results:**

The current study aimed to compare the output of 16S rRNA gene sequencing to the output of the quick IS-pro analysis, using vaginal swab samples from 297 women of reproductive age. 16S rRNA gene sequencing and IS-pro analyses yielded very similar vaginal microbiome profiles, with a median Pearson’s R^2^ of 0.97, indicating a high level of similarity between both techniques.

**Conclusions:**

We conclude that the results of 16S rRNA gene sequencing and IS-pro are highly comparable and that both can be used to accurately determine the vaginal microbiota composition, with the IS-pro analysis having the benefit of rapidity.

**Supplementary Information:**

The online version contains supplementary material available at 10.1186/s12866-021-02149-7.

## Background

The presence and composition of human microbiota have received increasing attention over the past decade. Although links between microbiota and host health and disease have been suggested for a long time [1, 2], the introduction of advances such as 16S rRNA gene sequencing has only recently made it possible to properly characterize an individual’s microbiome. While microbiome research has initially focused on the gut microbiome, it has become clear that also the vaginal microbiome is highly relevant for women’s health [3].

Previous studies have shown that the vagina of healthy women is usually dominantly colonized by a large amount of one out of a limited number of different Lactobacilli. The four most common of Lactobacilli dominant vaginal microbiome profiles are characterized by either *L. iners*, *L. crispatus*, *L. gasseri*, or *L. jensenii* [4]. These commensal Lactobacilli decrease the vaginal pH through production of lactic acid, which is thought to provide an acidic barrier to opportunistic pathogens. However, not all women have a *Lactobacillus* dominant vaginal flora. Importantly, a non-Lactobacilli dominated profile is associated with the clinical condition of bacterial vaginosis (BV) [5].

BV is the most common vaginal disorder in women and occurs in up to 20% of pregnant women [6]. In BV, the overgrowth of typically non-Lactobacillus anaerobic bacteria, such as *Gardnerella vaginalis*, *Mobiluncus spp*., and *Atopobium vaginae,* leads to a disruption of the ecological vaginal balance [7] and an alteration of the vaginal milieu, which may give rise to clinical symptoms such as itchiness and vaginal discharge [7, 8]. However, roughly 50% of the women who have BV are asymptomatic or have less obvious symptoms [9].

Accurate profiling of the microbiota is becoming a highly important tool for diagnosis and potential prediction for a range of clinical phenomena, such as premature delivery based on vaginal microbiota composition or diverticulitis with gut microbiota composition [10, 11]. Currently, 16S rRNA gene sequencing is seen as the gold standard of obtaining microbiota profiles. 16S rRNA gene sequencing, however, is still a relatively expensive and more importantly, labor intensive procedure, making cheaper and faster alternatives imperative. From this perspective, Budding et al. [12] developed a new PCR based profiling technique for analysis of complex microbiota, namely the intestinal microbiota.

**Fig. 1:**
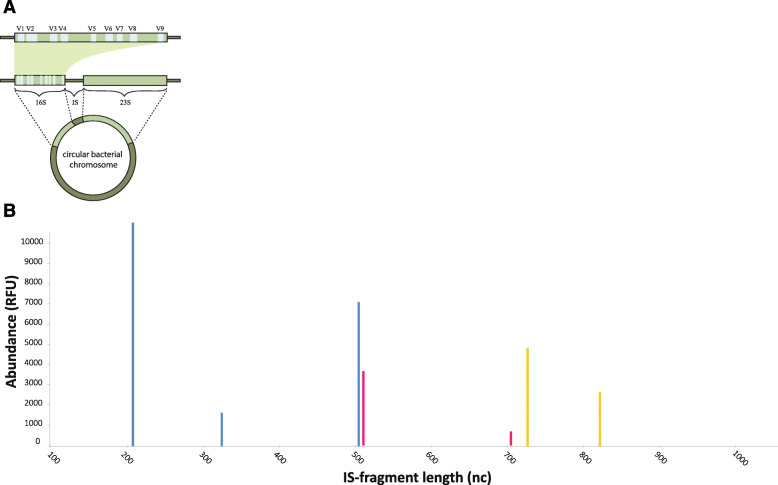
**a** A representation of the circular chromosome of bacteria. The 16S and 23S ribosomal RNA genes are highlighted together with the intergenic space (IS) region. **b** IS profile of vaginal swab. Peak length, expressed in nucleotides, corresponds to IS-fragment length. Peak height, expressed in relative fluorescence units (RFU), reflects quantity of fragments. Red peaks represent *Bacteroidetes*, yellow peaks represent *Proteobacteria*, blue peaks represent *FAFV*

IS-pro detects by their DNA, in particular a universal ribosomal DNA region which is unique for each bacterial species: the 16S–23S rRNA intergenic spacer (IS) region (Fig. 1a). IS-pro combines bacterial differentiation by the length of the IS region with instant taxonomic classification by phylum-specific fluorescent labeling of PCR primers, which bind to phylum specific regions in the 16S rRNA (Fig. 1b). These combined parameters can resolve bacterial taxa to the species level [12]. Three groups of bacterial phyla are differentiated: *Bacteroidetes*, *Proteobacteria* and a combination of the phyla *Firmicutes, Actinobacteria, Fusobacteria* and *Verrucomicrobia* (FAFV group). The IS region is extremely variable in size and sequence compared to the 16S region, even with closely related taxonomic groups [13], making it more suitable for analysis of complex communities.

IS-pro has advantages compared to next-generation sequencing (NGS) approaches. IS-pro has only a limited sample processing steps, consisting of DNA isolation, PCR and capillary electrophoresis (CE). There is no need for quantification or purification of DNA, no need for ligation of adapters or any of the other time-consuming steps needed for 16S-sequencing. This renders the technique easily implementable and results in a fast turnaround time, of approximately 4 h from sample collection to analyzed results. Furthermore, IS-pro has lower costs, as DNA isolation and PCR are needed for both IS-pro and 16S sequencing, but for IS-pro only an additional capillary electrophoresis step is needed, whereas 16S sequencing requires a number of additional preprocessing steps and the sequencing step, which is orders of magnitudes more costly than CE. Of course, high costs of sequencing can be mitigated by processing of large batches. However, processing of smaller batches may be more attractive for many settings, such as clinical diagnostics or research with small sample numbers [14]. Finally, the IS-pro technique has a high level of standardization and is able to process samples with low bacterial load very efficiently.

In this study we compared a sample-per sample microbiota analysis by Illumina-based 16S rRNA gene sequencing to IS-pro analysis on vaginal swabs taken from women prior to the start of IVF or IVF-ICSI treatment.

## Results

### 16S rRNA gene sequencing quality control

After sequencing of the vaginal samples, all reads were monitored for quality control purposes. The quality criteria are described in the Methods section below.

A total of 294 (of 297) vaginal samples produced sequences matching the quality criteria. Sequencing of the microbial DNA collected by the vaginal swabs resulted in 17.947.706 reads of which 8.374.321 reads passed quality control and could be assigned to a taxon. Vaginal samples yielded a median of 9.661 reads per sample. During the OTU calling, a minimum of 100 reads were used for taxonomic assignment, leading to the assignment of 75 species and 22 genera to the samples with an average of 29 (standard deviation 21,7) assigned species or genera per vaginal sample. Two negative controls yielded on average 9 taxonomically classified reads after processing. Resulting distributions of assigned taxa can be seen in Table S1.

### 16S rRNA gene sequencing results of vaginal samples

**Fig. 2:**
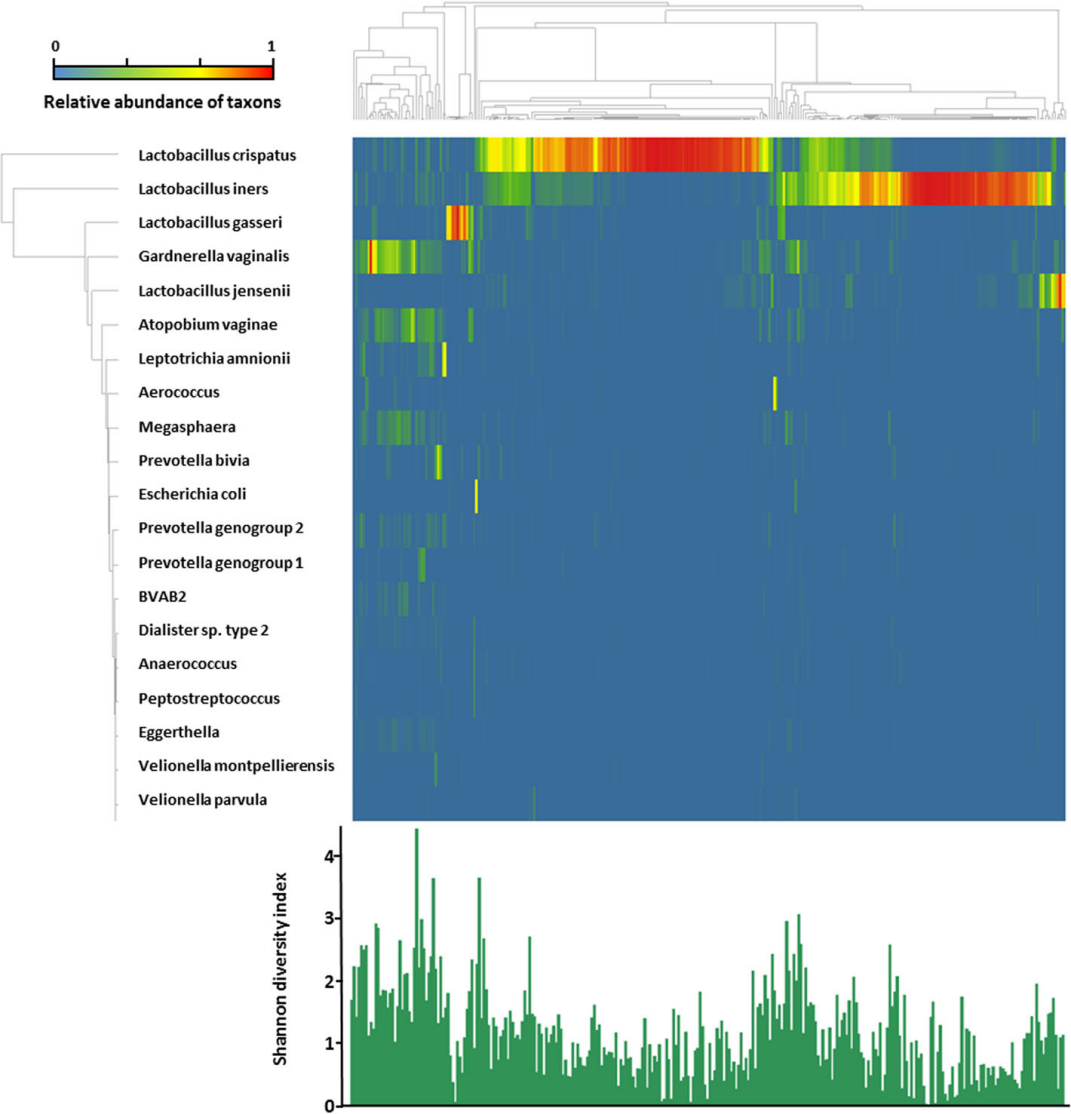
Heatmap of relative microbiome abundance found in vaginal samples obtained from 294 women through 16S rRNA gene sequencing. Column correlation clustering was performed with the UPGMA method to cluster microbiome profiles according to similarity based on cosine correlation. Row hierarchy clustering was performed calculating the Euclidean distance based on hierarchical analysis to identify and order the most prominent taxa related to the microbiome profiles. Shown in the figure are the 20 most abundant taxa found in this correlation. The alpha diversity is shown in the bar graph using the Shannon diversity index in a sample order correlating with the above heatmap profiles

The heatmap in Fig. 2 shows the microbiota profiles of 294 vaginal samples, displaying relative abundance of bacterial taxa, clustered based on cosine correlation. As has been described in other studies, the Shannon diversity index of samples that are not dominated by a single *Lactobacillus* species generally show an increased microbial diversity.

Table 1 (columns) shows the hierarchical clustering of the vaginal microbiome profiles analyzed by 16S rRNA gene sequencing. The most common found cluster was a *L. crispatus* dominant cluster in 44.9% (132/294) of the samples. The cluster ‘Diverse’ includes all vaginal samples which do not show dominance of a single *Lactobacillus* species, and commonly include *Gardnerella vaginalis*. The vaginal samples clustered in ‘Other’ were characterized by a number of non-*Lactobacillus* bacteria, e.g. *Leptotrichia* or *Prevotella*, and could not be ascribed to any specific cluster.

**Table 1:** Distribution of vaginal sample cluster profiles between 16S rRNA gene sequencing results and IS-pro results, respectively. Only samples successfully analyzed by both techniques are shown. It is expressed in percentage how many profiles with IS-pro matched the profile analyzed with 16S sequencing

	16S rRNA gene sequencing vaginal profiles (***n*** = 294)						
	Cluster	Diverse *n* = 38	*L. crispatus* *n* = 132	*L. gasseri* *n* = 17	*L. iners* *n* = 74	*L. jensenii* *n* = 22	Other *n* = 11
**IS-pro vaginal profiles (*n*** **= 297)**	Diverse *n* = 12	6 (15.8%)	2	2			
	*L. crispatus* *n* = 133	4	113 (85.6%)	3	6	3	3
	*L. gasseri* *n* = 14	4	1	8 (47.1%)			1
	*L. iners* *n* = 129	21	16	4	68 (91.9%)	13	7
	*L. jensenii* *n* = 7	1				6 (27.3%)	
	Other *n* = 2	2					

### IS-pro results of vaginal samples

**Fig. 3:**
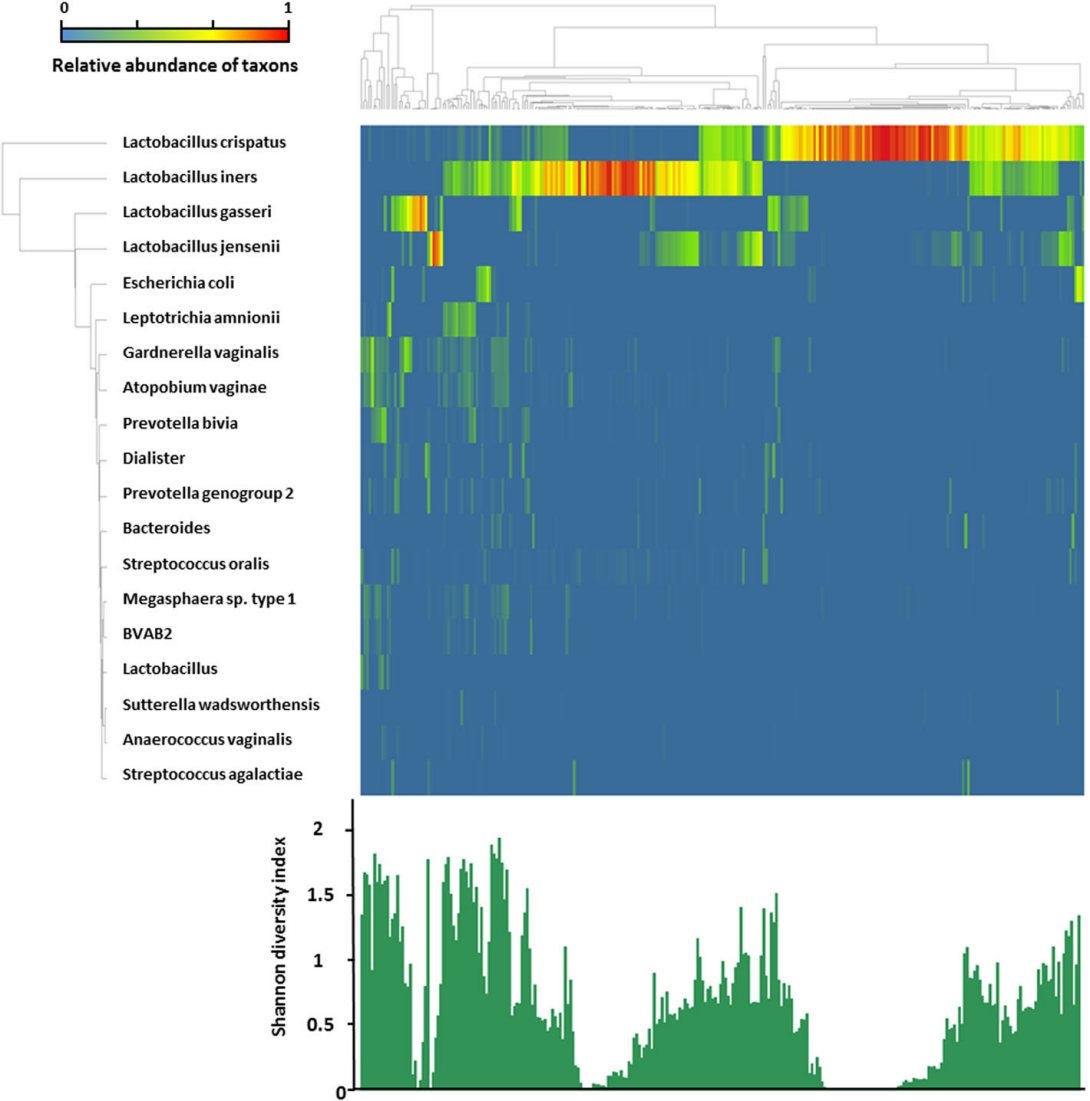
Heatmap of relative microbiome abundance found in vaginal samples obtained from 297 women with IS-pro. Column correlation clustering was performed with the UPGMA method to cluster microbiome profiles according to similarity based on cosine correlation. Row hierarchy clustering was performed calculating the Euclidean distance based on hierarchical analysis to identify and order the most prominent taxa related to the microbiome profiles. Shown in the figure are the 19 most abundant taxa found in this correlation. The alpha diversity is shown in the bar graph using the Shannon diversity index in a sample order correlating with the above heatmap profiles

Of the 297 vaginal samples, all samples passed the quality control as described in the Methods. The heatmap in Fig. 3 shows the microbiome profiles of the 297 vaginal samples characterized with IS-pro, displaying relative abundance of bacterial species clustered based on cosine correlation. Distributions of bacterial taxa based on IS-pro can be found in Table S2.

Table 1 (rows) shows the hierarchical clustering of the vaginal microbiome profiles analyzed by IS-pro. As with the analysis with 16S, the most common found cluster in IS-pro results was also a *L. crispatus* dominant cluster 44.8% (133/297).

### Comparison of 16S rRNA gene sequencing and IS-pro profiles of vaginal samples

**Fig. 4:**
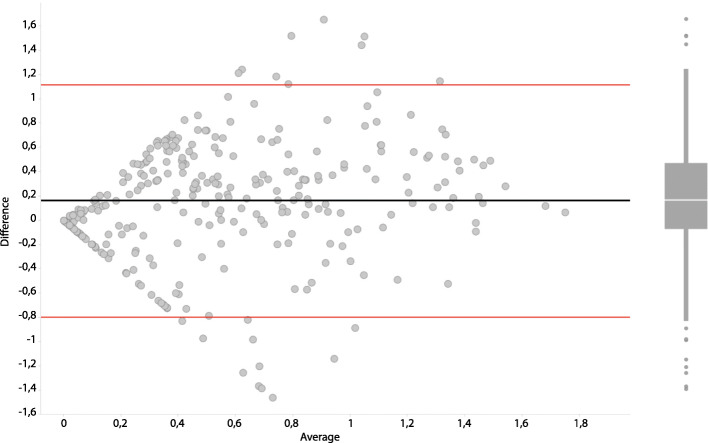
Bland Altman plot of the Shannon indices derived from the microbiota profiles gathered through both the 16S rRNA gene sequencing and IS-pro analyses. In it the average Shannon index between 16S rRNA gene sequencing and IS-pro analyses outcomes of matched samples is plotted against the difference between these outcomes. Red lines indicate the 95% Confidence Interval of the Limits of Agreement. The boxplot indicates the median and interquartile ranges of the number of datapoints in the Bland Altman plot

A Bland Altman analysis of the Shannon indices from 16S rRNA gene sequencing and IS-pro analyses shows that the vast majority of microbiota profiles have a similar Shannon diversity index (Fig. 4). A larger difference indicates a higher diversity in the 16S rRNA gene sequencing data. Examination of the most dissimilar samples showed that dissimilarity was based on samples that were very dominated by one Lactobacillus species when analyzed with one technique, while showing a high diversity when analyzed with the other technique. Neither technique showed a consistent difference in diversity, suggesting that this is not a structural feature of either of the techniques that were used.

Table 1 shows a cross table depicting distribution of vaginal sample profile clusters between 16S rRNA gene sequencing and IS-pro. From this table it can be read which profiles yielded the same profile with both techniques and which profiles did not match. In percentages it is indicated how many profiles with IS-pro matched the profile analyzed with 16S sequencing. A large difference is found in the Diverse cluster, which only had 15.8% matching profiles compared to smaller differences of 85.6 and 91.9% for for *L. crispatus* and *L. iners*, respectively. This shows a meaningful overview of the comparability of the results between the two techniques used.

**Fig. 5:**
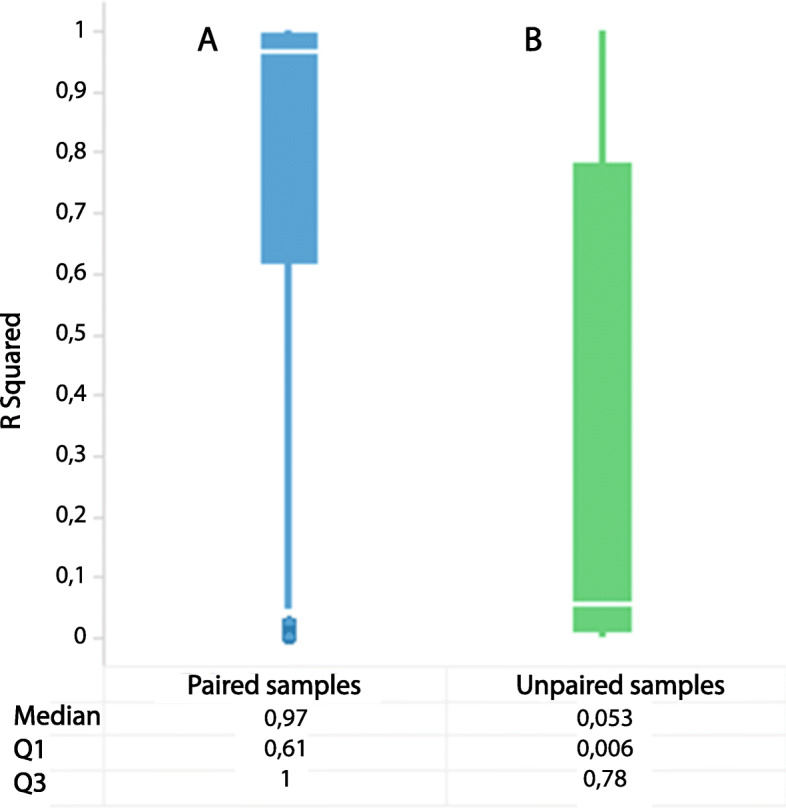
Outcomes of Pearson’s correlation (expressed as R2) where blue bars represent outcomes from analyses based on paired samples from the same patient, and green bars represent the same analyses where samples were not paired per patient. **a** & **b** Boxplot featuring R squared values of IS-pro vaginal sample outcomes correlated to those of 16S rRNA gene sequencing vaginal sample outcomes when samples are paired per patient (**a**) vs no pairing (**b**). (Q1 = 1st quartile, Q3 = 3rd quartile)

Both methods yielded almost completely comparable *L. crispatus* cluster assignments. Sixty-eight samples (out of the 294) were assigned to the *L. iners* cluster by both methods. To statistically determine the comparability of the microbiota compositions as determined by 16S rRNA gene sequencing and IS-pro, we calculated Pearson’s R correlation in paired samples from the same patient. This comparison showed a high correlation of the IS-pro and 16S rRNA gene sequencing results, with a median R^2^ of 0.97 (Fig. 5). In addition to the paired samples, Fig. 5 also shows a comparison to unpaired samples. In support of the fact that the paired samples not only have a high correlation due to good by chance, as they may consist of a simple microbiota dominated by a limited number of similar microbes.

**Fig. 6:**
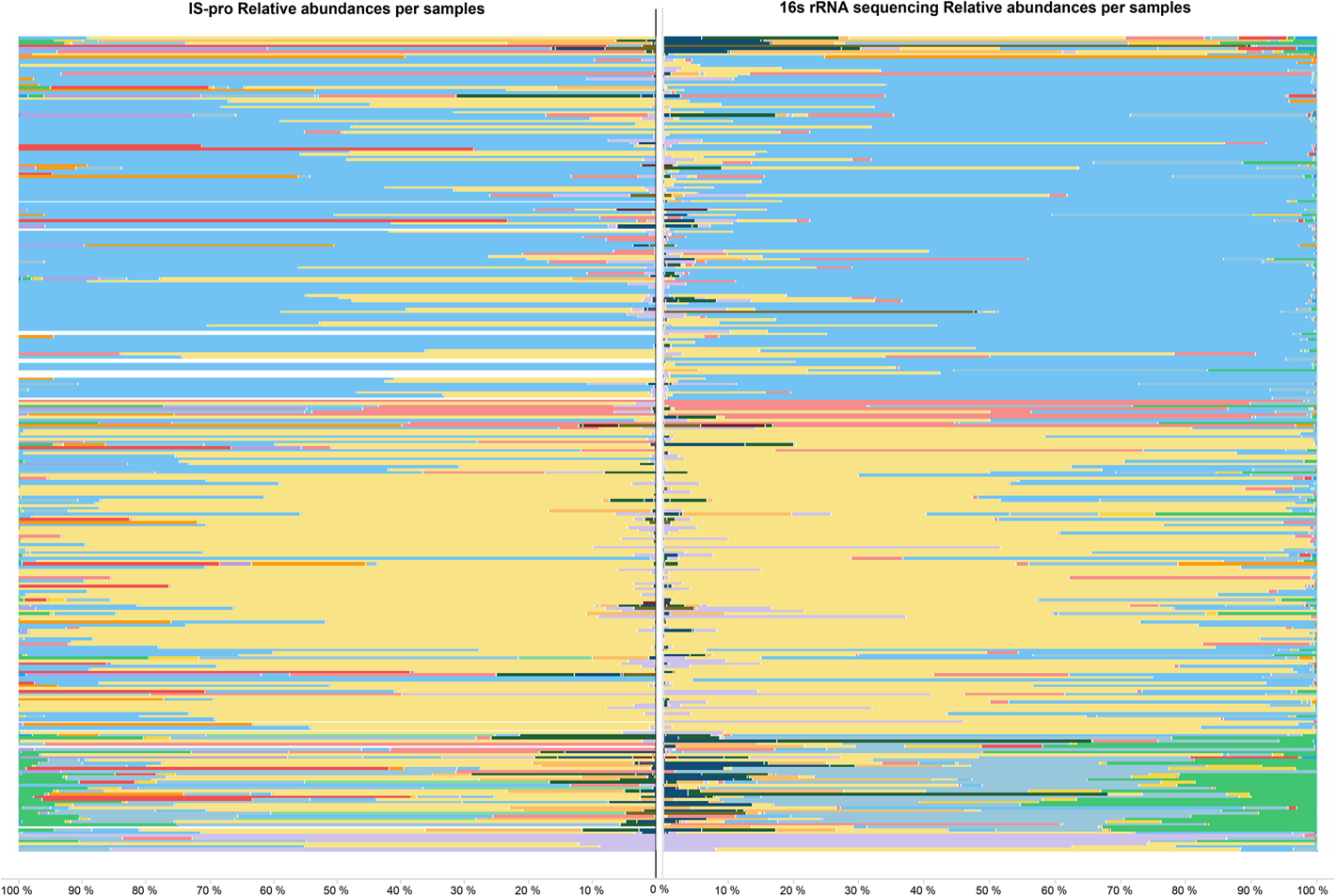
Graph showing the relative abundances in the microbiota profiles gathered through IS-pro (Left) and 16S rRNA gene sequencing (Right) analyses. Every line represents a single sample. Empty lines indicate failed analyses by one of the analysis techniques

In Fig. 6a graph is shown wherein the microbiota profiles obtained through both 16S rRNA sequencing and IS-pro analysis are placed side by side per sample for direct visual comparison of the whole dataset. The samples are seen to be highly similar to each other.

## Discussion

16S rRNA gene sequencing is currently seen as the gold standard for the profiling of the microbiome. However, for routine diagnostics or rapid processing of (small) sample batches, 16S rRNA gene sequencing is not well suited due to costs and time-consumption, creating space for other techniques.

In this study we show that vaginal microbiome profiling using the quick and non-expensive IS-pro technique creates outcomes highly comparable to those of the 16S rRNA gene sequencing, highlighting that the IS-pro technique can serve as an accurate test method for microbiome profiling, as was also demonstrated in a previous study [12].

Although the IS-pro and 16S rRNA analyses in our study were highly comparable, a number of practical differences were observed, which may be significant depending on the specific needs of a researcher. Microbiome profiling through 16S rRNA gene sequencing involved the use of a large number of bio-informatics tools and had high costs. The IS-pro technique was developed with the goals of cost-effectiveness and simplicity in mind. In our study, the laboratory processing of the sample as well as the data-analysis were completed faster than the 16S rRNA gene sequencing analysis, and at reduced cost.

The results of the vaginal profiling using both 16S rRNA gene sequencing and IS-pro were found to be highly similar. However, when looking at the clustering of samples based on the similarity of profiles, there are still differences in the number of samples per cluster, especially in the more diverse clusters. The differences are generally caused by small differences in abundance of species in profiles per technique. Since clusters were formed by performing a UPMGA clustering on a cosine correlation matrix, small differences between samples may lead to different clustering outcomes. Importantly, the Bland Altman analysis shows that the vast majority of samples fall within the 95% Confidence Interval of the Limits of Agreement, with the exception of a set of 20. Although all these samples are dominated by *Lactobacilli,* depending on the technique used the assigned species is either *L. iners* or *L. crispatus*. The difference indicates improvement is needed in regard to determine the correct species and with it, the correct assignment of species between the techniques.

A strength of the study is the focus of analyses on the vaginal microbiota. The vagina allows for analyses of a bacterial niche in which most taxa can be identified to the species level allowing proper comparison of output quality between the techniques. Additionally, the large number of samples which has been analyzed strengthens the statistical analyses.

A limitation of the study is the difference in databases used for the 16S rRNA gene sequencing and IS-pro data processing, which did not completely overlap, and hereby perhaps having missed bacterial species in either technique. Finally, it is possible that potential PCR bias plays a more significant role in the 16S rRNA gene sequencing procedure than in the IS-pro analysis as the former entails two sequential PCR procedures amplifying the target DNA, leading to more distortion or bias of outcomes.

## Conclusions

In conclusion, 16S rRNA gene sequencing and IS-pro analysis produce highly comparable results when analyzing vaginal microbiota collected by swabs. IS-pro analysis has the potential to increase speed and reduce costs of these analyses while maintaining the same quality of the profiling, allowing to accelerate research into the vaginal microbiome and opening the possibility of using vaginal microbiota profiling as a rapid diagnostic tool.

## Methods

### Sampling

Two hundred ninety-seven women attending eight different reproductive health clinics for IVF or IVF/ICSI treatment in the Netherlands were included in this prospective study. The medical ethics testing committee Erasmus MC has approved the ethicality of the study under reference MEC-2014-455. All the procedures and methods have been performed according to the guidelines of the ethics committee and in accordance with local laws and regulations. All participants were informed of the study contents and signed an informed consent form before inclusion into the study. Vaginal swabs were self-collected at one of eight participating clinics from June 2015 until March 2016. The vaginal swabs were self-collected at the clinics and directly placed in 0.5 ml of reduced transport fluid (RTF, Microbiome, Amsterdam, The Netherlands) at 2-8 °C for a maximum of 2 h, after which the swab is stored at -20 °C. Samples were transferred on dry ice and stored at -20 °C until further processing. The same DNA, extracted from a single vaginal sample of each participant was used for both analysis techniques.

### DNA extraction and sample preparation

DNA was extracted from vaginal swab suspensions with the Chemagen (Perkin-Elmer, Baesweiler, Germany) automated DNA extraction machine using the buccal swab extraction kit according to the manufacturer’s instructions. In short, swab suspensions were thawed and vortexed. Two hundred microliters of sample was incubated with 200 μl Chemagen lysis buffer and 10 μl Proteinase K at 56 °C while shaking at 500 rpm. DNA was extracted with the protocol buccal Swab Prefilling. Elution of DNA was in 100 μl of Chemagen Elution buffer.

### Formation of the library

Sample DNA concentration was measured with the Picogreen dsDNA assay (Thermofisher, MA, USA). A PCR amplifying the V3/V4 region of the 16S rRNA gene region was performed with individually distinguishable dual index primer sets, which were developed to distinguish low diversity microbiomes on each sample as has previously been described by Fadrosh et al. [15]. The universal primer set 319F/806R, altered to also encode the Illumina sequencing primer and barcode labelling sequences, was used during the PCR. PCR conditions were as follows: 30 s at 98 °C, then 30 cycles of 10 s at 98 °C, 15 s at 58 °C, and 15 s at 72 °C and a final step of 3 min at 72 °C.

The amplified DNA was purified with the AMPure XP magnetic bead assay (BeckmanCoulter Genomics, Danvers, MA, USA) quantified as above, recalculated into nM with the formula: “[nM DNA] = DNA concentration (ng/μl) x 1e6 (μl/L) / (Sample fragment size in bp x 656,4 (g/mole))” and equalized to 12 nM. To ensure quality, pooled DNA that did not reach at least 8 nM was not used for 16S rRNA gene sequencing analysis.

### 16S rRNA gene sequencing

16S rRNA gene sequencing of the pooled samples was performed by the Tumor Genome Analysis Core group of the Department of Pathology at the Amsterdam UMC, location VUmc in Amsterdam, The Netherlands with a Miseq tabletop sequencer (Illumina, San Diego, CA, USA).

### Sequencing data analysis

Data generated through the 16S rRNA gene sequencing was processed with QIIME to remove primer and index sequences. A minimum Phred quality score threshold of 5 was upheld throughout the processing. Paired end reads with no errors in the barcode matching, a minimum overlap of six nucleotides, and a minimum combined length of 400 nucleotides were assembled to produce identifiable sequences. Operational Taxonomic Units (OTU) were picked with the Usearch method [16]. During this process the sequences were sorted based on length and abundance of identical reads, checked for chimeric sequences, and clustered at 97% identity to denoise the data. These OTUs were aligned to the reference database with the PyNAST method for sequence alignment and subsequently assigned with the RDP classifier method which uses a Naïve Bayes classification. The assignment of OTUs was performed by using the database previously described by Srinivasan et al. [17], assigning sequences on a genus to species level. The remaining sequences were BLASTed, and included if the sequence in question could be identified at a genus or species level.

### Intergenic spacer profiling (IS-pro)

Amplification of 16S–23S rRNA intergenic spacer (IS)-regions was performed with the IS-pro assay (InBiome B.V., Amsterdam, The Netherlands). IS-pro differentiates bacterial species by the length of the 16S–23S rRNA IS-region with taxonomic classification by phylum-specific fluorescently labeled PCR primers [12]. The assay consists of two multiplex PCRs: one PCR contains two different fluorescently-labeled primers: one for the phyla *Actinobacteria, Firmicutes, Fusobacteria* and *Verrucomicrobia* and a second color for the phylum *Bacteroidetes*. A separate PCR is performed for the phylum *Proteobacteria.* The assay was performed according to the protocol provided by the manufacturer. Amplifications were carried out on a GeneAmp PCR system 9700 (Applied Biosystems, Foster City, CA). After PCR, 5 μl of PCR product was mixed with 20 μl formamide and 0.5 μl Mapmaker 1500 ROX-labeled size marker (BioVentures, Murfreesboro, TN, USA). DNA fragment analysis was performed on an ABI Prism 3500 Genetic Analyzer (Applied Biosystems). Species were assigned to peaks by using a database compiled of IS-pro fragments obtained from in-silico and in vitro IS-pro PCRs of known vagina associated bacterial species. An internal amplification control (IAC) was used to control the PCR reaction for inhibition. A sample passed the quality control when the IAC signal was present in sufficient amount (3 of 5 IAC peaks > 500 Relative Fluoresence Units (RFU)) or when a sufficiently high bacterial signal was present (at least one bacterial peak > 20.000 RFU).

### Data analysis

Alpha diversity of the microbiome per sample was measured by calculating the Shannon diversity index of individual samples. The Shannon indices per samples were used for a Bland Altman method comparison using Graphpad Prism 8 (GraphPad Software, La Jolla California USA). 95% limits of Agreement were set at 1,96 time the standard deviation, which was the default. Relative abundance of microbiome per sample was used to perform a correlation clustering of all sample profiles according to the UPGMA method. Relative abundance for 16S rRNA gene sequencing data was calculated as a percentage of reads from total reads; for IS-pro, relative abundance is given as fluorescence intensity per peak as a percentage of total fluorescence. This data was then used to identify the major clusters making up both the datasets. Pearson’s R linear regression was used to compare abundance of species between samples. R^2^ values were used to show the percentage variation of microbiota. For Pearson’s R calculations only species that were available in both the 16S rRNA gene sequencing and IS-pro databases were included.

## Supplementary Information

**Additional file 1: Supplemental data table 1.** Output from the 16s rRNA gene sequencing in Vagina samples. **Supplemental data table 2.** Output from the IS-pro analysis per sample.

## Data Availability

The datasets generated and/or analyzed during the current study are available in the SRA database repository, https://www.ncbi.nlm.nih.gov/sra/SRP133380 . The database that supports the IS-pro findings of this study are available from InBiome B.V.. but restrictions apply to the availability of these data, which were used under license for the current study, and so are not publicly available. Data are however available from the authors upon reasonable request and with permission of InBiome B.V..

## Abbreviations

BV: Bacterial vaginosis
DNA: Deoxyribonucleic acid
FAFV: Firmicutes, Actinobacteria, Fusobacteria and Verrucomicrobia (FAFV group)
IS: Intergenic spacer
IAC: Internal amplification control
ICSI: Intracytoplasmic sperm injection
IVF: In vitro fertilization
NGS: Next-generation sequencing
OTU: Operational Taxonomic Units
PCR: Polymerase chain reaction
RFU: Relative Fluoresence Units
rRNA: Ribosomal RNA
RTF: Reduced transport fluid
UPGMA: Unweighted pair group method with arithmetic mean
